# Pilot implementation of rural rehabilitation services, India

**DOI:** 10.2471/BLT.22.288168

**Published:** 2022-09-19

**Authors:** Rajani Mullerpatan, Prasad Waingankar, Shrutika Parab, Bela Agarwal, Omeshree Nagrale, Shashank Dalvi

**Affiliations:** a Mahatma Gandhi Mission School of Physiotherapy, Mahatma Gandhi Mission Institute of Health Sciences, Sector 1, Kamothe, Navi Mumbai-410209, Maharashtra, India.; b Department of Community Medicine, Mahatma Gandhi Mission Institute of Health Sciences, Navi Mumbai, India.

## Abstract

**Objective:**

To implement rehabilitation services in a rural area of Raigad district, Maharashtra, India.

**Methods:**

We piloted a rehabilitation service delivery model through the Mahatma Gandhi Mission Institute of Health Sciences, in five villages. The institute performed participatory rural appraisal and focus group discussions with stakeholders to identify general issues in village life that could affect delivery. To integrate rehabilitation service delivery into the existing primary care system, a team from the institute developed a strategic plan through multidisciplinary clinical meetings. A rehabilitation team conducted a door-to-door survey and referred people needing rehabilitation services to the outreach visits the team was making to the primary health centre twice a week. If needed, patients could be referred to a university teaching hospital for tertiary-level care.

**Findings:**

The rural appraisal identified lack of awareness, inadequate workforce and infrastructure as key issues for rehabilitation services delivery. In response, we conducted awareness campaigns and formed a rehabilitation team consisting of personnel and students from the institute. Between 2018 and 2021, the team provided care to 1800 patients, of which half (900 patients) had musculoskeletal disorders. After rehabilitation, 360 (40%) of these 900 patients performed daily-living activities and continued to work with reduced pain within 2–3 days after rehabilitation. The team provided antenatal care to 1629 pregnant women with musculoskeletal pain or stress urinary incontinence.

**Conclusion:**

Provision of rehabilitation services built awareness about physiotherapy, developed a rehabilitation care pathway and established a need for regular services. Using existing resources of the institute and involving students rendered the model sustainable.

## Résumé

**Objectif:**

Proposer des services de réadaptation dans une région rurale du district de Raigad, dans le Maharashtra, en Inde.

**Méthodes:**

Nous avons déployé un modèle de prestation de services de réadaptation dans cinq villages par l'intermédiaire du Mahatma Gandhi Mission Institute of Health Sciences. L'institut a procédé à une évaluation rurale participative et organisé des discussions au sein de groupes de réflexion impliquant différentes parties prenantes, afin de repérer les principaux problèmes rencontrés dans les villages et susceptibles d'affecter les prestations. En vue d'intégrer les services de réadaptation dans le système de soins primaires existant, une équipe de l'institut a élaboré un plan stratégique lors de réunions cliniques multidisciplinaires. De son côté, une équipe de réadaptation a effectué une enquête en porte-à-porte et proposé aux personnes nécessitant de tels services de se rendre dans le centre de soins de santé primaires, à l'occasion des visites de proximité qu'elle réalise deux fois par semaine. Si besoin, les patients pouvaient être transférés vers un hôpital universitaire pour des soins de niveau tertiaire.

**Résultats:**

L'évaluation rurale a permis d'identifier les obstacles majeurs à la prestation de services de réadaptation: un manque de sensibilisation, ainsi que l'absence d'infrastructures et de personnel qualifié. Pour y remédier, nous avons mené des campagnes de sensibilisation et formé une équipe de réadaptation, composée d'employés et d'étudiants de l'institut. Entre 2018 et 2021, cette équipe a pris en charge 1800 patients; la moitié (900 patients) souffraient de troubles musculosquelettiques. À l'issue de leur réadaptation, 360 (40%) de ces 900 patients étaient capables d'accomplir les tâches du quotidien et ont repris le travail 2 à 3 jours plus tard avec une douleur moins vive. L'équipe a également prodigué des soins prénatals à 1629 femmes enceintes présentant des douleurs musculosquelettiques ou une incontinence urinaire à l'effort.

**Conclusion:**

Les prestations de réadaptation ont permis de faire connaître la kinésithérapie, de développer un parcours de soins en la matière et d'exprimer la nécessité d'une régularité des services. L'utilisation des ressources de l'institut et l'implication des étudiants ont assuré la viabilité du modèle.

## Resumen

**Objetivo:**

Establecer servicios de rehabilitación en una zona rural del distrito de Raigad, Maharashtra, en la India.

**Métodos:**

Se puso a prueba un modelo de prestación de servicios de rehabilitación a través de Mahatma Gandhi Mission Institute of Health Sciences en cinco pueblos. El instituto llevó a cabo una evaluación rural participativa y debates en grupos de discusión con las partes interesadas para identificar los problemas generales en la vivencia del pueblo que podrían afectar a la prestación. Para integrar la prestación de servicios de rehabilitación en el sistema de atención primaria existente, un equipo del instituto elaboró un plan estratégico mediante reuniones clínicas multidisciplinares. Un equipo de rehabilitación realizó una encuesta puerta a puerta y remitió a las personas que necesitaban servicios de rehabilitación a las visitas de promoción que el equipo realizaba al centro sanitario primario dos veces por semana. En caso necesario, se podía derivar a los pacientes a un hospital universitario para que recibieran atención de nivel terciario.

**Resultados:**

La evaluación rural identificó la falta de concienciación, la falta de personal y los problemas de infraestructura como problemas clave para la prestación de servicios de rehabilitación. En respuesta, se organizaron campañas de concienciación y se formó un equipo de rehabilitación compuesto por personal y estudiantes del instituto. Entre 2018 y 2021, el equipo prestó atención a 1800 pacientes, de los que la mitad (900 pacientes) tenían trastornos osteomusculares. Después de la rehabilitación, 360 (40 %) de estos 900 pacientes realizaron actividades de la vida diaria y siguieron trabajando con un dolor reducido en los 2 a 3 días posteriores a la rehabilitación. El equipo prestó asistencia prenatal a 1629 mujeres embarazadas con dolor osteomuscular o incontinencia urinaria de esfuerzo.

**Conclusión:**

La prestación de servicios de rehabilitación permitió concienciar sobre la fisioterapia, desarrollar una vía de atención a la rehabilitación y establecer la necesidad de servicios regulares. La utilización de los recursos existentes en el instituto y la participación de los estudiantes hicieron que el modelo fuera sostenible.

## ملخص

**الغرض:**

تنفيذ خدمات إعادة التأهيل في منطقة ريفية في منطقة رايجاد، ماهاراشترا، بالهند.

**الطريقة:**

قمنا بتجريب نموذج تقديم خدمة إعادة التأهيل من خلال معهد مهاتما غاندي لبعثة العلوم الصحية في خمس قرى. أجرى المعهد تقييمًا ريفيًا مشتركًا، ومناقشات جماعية مركزة مع أصحاب المصلحة لتحديد القضايا العامة في حياة القرية، والتي بإمكانها أن تؤثر على تقديم الخدمة. لدمج تقديم خدمة إعادة التأهيل في نظام الرعاية الأولية القائم، قام فريق من المعهد بوضع خطة إستراتيجية من خلال اجتماعات تحليلية متعددة التخصصات. قام فريق إعادة التأهيل بإجراء استطلاع للرأي من خلال الزيارات المنزلية، وإحالة الأشخاص الذين يحتاجون إلى خدمات إعادة التأهيل إلى زيارات التوعية التي كان يقوم بها الفريق إلى مركز الصحة الأولية مرتين في الأسبوع. وإذا لزم الأمر، يمكن إحالة المرضى إلى مستشفى تعليمي جامعي للحصول على رعاية من المستوى الثالث.

**النتائج:**

أشار التقييم الريفي للنقص في الوعي، وعدم ملاءمة قوى العمل، وضعف البنية التحتية، باعتبارها قضايا رئيسية لتقديم خدمات إعادة التأهيل. وكاستجابة لذلك، قمنا بحملات توعية وشكلنا فريقًا لإعادة التأهيل يتكون من موظفين وطلاب من المعهد. قام الفريق بين عامي 2018 و2021، بتقديم الرعاية لعدد 1800 مريض، يعاني نصفهم (900 مريض) من اضطرابات عضلية هيكلية. بعد إعادة التأهيل، قام 360 (%40) من هؤلاء المرضى البالغ عددهم 900 بأداء أنشطة الحياة اليومية، واستمروا في العمل مع تخفيف الألم في غضون 2 إلى 3 أيام بعد إعادة التأهيل. قدم الفريق رعاية ما قبل الولادة لعدد 1629 امرأة حامل يعانين من آلام عضلية هيكلية، أو سلس البول الإجهادي.

**الاستنتاج:**

أدى تقديم خدمات إعادة التأهيل إلى بناء الوعي بالعلاج الطبيعي، ووضع مسار لرعاية إعادة التأهيل، وإنشاء الحاجة إلى الخدمات المنتظمة. أدى استخدام الموارد الحالية للمعهد وإشراك الطلاب إلى جعل النموذج مستدامًا.

## 摘要

**目的:**

在印度马哈拉施特拉邦莱加德农村地区提供康复服务。

**方法:**

我们通过圣雄甘地医学健康科学研究所，在五个村庄开展康复服务试点。该研究所与利益攸关方进行了参与式农村评估和专题小组讨论，确定了农村生活中可能影响康复服务的一般问题。为了将康复服务整合到现有的初级医疗保健体系中，由研究所人员组成的小组在临床应用多学科会议上制定了一项战略计划。我们的一个康复小组进行了挨家挨户的调查，将需要康复治疗的患者转介到外展服务中心，该小组每周前往初级卫生保健中心进行两次会诊。如需要，会将患者转介到医学院的附属医院进行三级护理。

**结果:**

农村评估发现，缺乏康复宣传、康复服务人员以及基础设施建设，这是提供康复服务面临的关键问题。因此，我们开展了宣传活动，并组建了一个由研究所人员和学生组成的康复小组。2018 年至 2021 年间，小组为 1800 名患者提供了护理服务，其中半数患者（900 名患者）患有肌肉骨骼疾病。康复治疗后，这 900 名患者中的 360 (40%) 名患者可以进行日常活动，并在康复后 2-3 天内疼痛减轻，继续开始工作。小组为 1,629 名患有肌肉骨骼疼痛或压力性尿失禁的孕妇提供了产前护理。

**结论:**

康复服务的提供促进了人们对物理治疗的认识，制定了康复护理方案并满足了人们对常规服务的需求。利用研究所的现有资源，并让学生参与，可以有效促进该模型的可持续性。

## Резюме

**Цель:**

Внедрение реабилитационных услуг в сельской местности района Райгад, штат Махараштра, Индия.

**Методы:**

При содействии Института медицинских наук имени Махатмы Ганди в пяти деревнях была экспериментально запущена модель предоставления реабилитационных услуг. Институт провел в сельской местности совместную оценку и обсуждения в фокус-группах с заинтересованными сторонами с целью выявления общих проблем в жизни деревни, которые могут повлиять на предоставление услуг. Чтобы интегрировать предоставление реабилитационных услуг в существующую систему первичной медико-санитарной помощи, командой института был разработан стратегический план в рамках междисциплинарных клинических совещаний. Группа по реабилитации провела подомовой опрос и направила людей, нуждающихся в реабилитационных услугах, на консультации в центр первичной медико-санитарной помощи, которые проводились два раза в неделю. При необходимости пациенты могли быть направлены в клиническую больницу при институте для получения специализированного медицинского обслуживания.

**Результаты:**

По результатам оценки, проведенной в сельской местности, ключевыми проблемами для предоставления реабилитационных услуг были признаны недостаточная осведомленность, нехватка рабочей силы и инфраструктуры. В ответ были проведены информационно-просветительские кампании и сформирована группа по реабилитации, состоящая из сотрудников и студентов института. В период с 2018 по 2021 год группа оказала помощь 1800 пациентам, из которых у половины (900 пациентов) наблюдались заболевания опорно-двигательного аппарата. После реабилитации 360 (40%) из этих 900 пациентов выполняли повседневную деятельность и продолжали работать с уменьшением боли в течение 2–3 дней после реабилитации. Группа предоставила дородовое наблюдение 1629 беременным женщинам с мышечно-скелетной болью или стрессовым недержанием мочи.

**Вывод:**

Предоставление реабилитационных услуг способствовало повышению осведомленности о физиотерапии, разработке плана реабилитации пациентов и формированию потребности в получении регулярных услуг. Использование существующих ресурсов института и привлечение студентов привели к устойчивости модели.

## Introduction

To achieve universal health coverage, rehabilitation needs to be integrated as an essential health service for people across their entire life-course. Although rehabilitation enhances quality of life and contributes to several sustainable development goals, rehabilitation services are under-prioritized within health-care systems in most parts of world, particularly in low- and middle-income countries.^1^

In middle-income countries, integrating rehabilitation services within health-care systems poses huge challenges because such services require multilevel and complex delivery models. For example, India’s large population, of which the majority reside in rural settings, stark diversity in socioeconomic conditions and inadequate resources challenge the authorities to deliver appropriate services. The pace and extent of integration of rehabilitation services into the health-care system do not meet the rising need for rehabilitation services due to lack of skilled workforce, infrastructure and resources.^2^^,^^3^ This gap exists despite the fact that India’s 2017 National Health Policy focuses on preventive and promotive health care and universal access to good quality health-care services with attention to disability and rehabilitation services.^4^

In the Raigad district, a rural area in Maharashtra state, the Mahatma Gandhi Mission Institute of Health Sciences – a higher education institute with an affiliated university teaching hospital – has provided community health-care services since 2008. Studies conducted by the institute between 2015 and 2018 showed poor knowledge of immunization; noncompliance with treatment of hypertension and diabetes; lack of trained workforce for newborn care; and elderly people experiencing poor health-related quality of life.^5^^–^^8^ These observations prompted integration of rehabilitation experts within the community medicine team to deliver comprehensive multidisciplinary rehabilitation services. Here we describe how a service delivery model for rehabilitation was implemented in the rural areas of Raigad district, using a community-based participatory approach.

## Methods

### Local setting

Raigad is the second largest district in Konkan region of Maharashtra state in India with a population of 2 634 200 (2011 census), of which 63% (1 659 546 people) live in rural areas.^9^ The district faces common rural health challenges, such as high prevalence of communicable diseases, malnutrition in children and elderly people and urinary incontinence in women.^10^

The public health system consists of one primary health centre at Nere village and a subcentre at Dhamani village. The health-care team at the primary health centre includes a medical officer, a resident auxiliary nurse, a midwife and a multipurpose worker. The Mahatma Gandhi Mission Institute of Health Sciences community medicine team consists of preventive-community medicine doctors and a medical social worker. This team is assisted by mobile community health workers (CHWs), including one accredited social health activist worker and *anganwadi* workers (that is, rural childcare workers). Before the implementation, the primary health centre and subcentre had no employed rehabilitation personnel.

For this pilot implementation, the Mahatma Gandhi Mission Institute of Health Sciences included a cluster of five villages with a total population of 4571 (Dhamani, Dhodani, Dehrang, Tawar Wadi and Waghachi Wadi) including its satellite habitats encompassing the entire Maldung *gram panchayat* of Panvel Tehsil. We selected these villages because the institute participated in the *Unnat Bharat Abhiyan* flagship scheme of the Indian government Ministry of Human Resource Development and adopted these villages through the Mahatma Gandhi Mission Community Health-Care Project.

### Participatory rural appraisal

In 2018, the community medicine department of the institute performed essential participatory rural appraisal in the villages. To identify general issues in village life, including health problems and other challenges, the community medicine team adopted a community need assessment approach. Villagers, including people living with disabilities, and accredited social health activist workers participated in informal discussions with the team during transect walks, and village mapping was conducted in all five villages. Transect walks were organized to follow the geographical map of each village. Village mapping was organized in an open space outside the *anganwadi* (rural childcare centre). One *anganwadi* worker, one youth volunteer and a few villagers participated in village mapping. The youth volunteer drew a rough sketch of the village map on the ground soil with a stick and ash. Thereafter, the detailed sketch was drawn on chart paper with a pencil, and coloured pens were used to mark critical landmarks in the village such as the *anganwadi*, *samaj mandir* (community hall), Dhamani subcentre and schools. 

Additionally, the community medicine team and a medical social worker held focus group discussions with pertinent stakeholders, namely: district officers, *gram panchayat* (council for a group of villages), a medical officer and a resident auxiliary nurse from the primary health centre, local leaders, primary school teachers and CHWs. Topics of discussion included health issues such as prevention of infectious diseases, noncommunicable diseases, breastfeeding, supplementary nutrition and anaemia.

### Service delivery model

To integrate rehabilitation service delivery into the existing primary care system, a team from Mahatma Gandhi Mission Institute of Health Sciences (including physiotherapists, preventive-community medicine doctors, nurses and medical social workers, along with members from obstetrics–gynaecology, paediatrics, medicine, otorhinolaryngology, ophthalmology, dermatology and orthopaedics departments) developed a strategic plan based on findings of the participatory rural appraisal. This team held monthly integrated multidisciplinary clinical meetings at the institute to discuss implementation of the strategies developed. During these meetings the following activities were undertaken: (i) designing of a comprehensive multidisciplinary rehabilitation framework; (ii) scheduling of monthly visits to the primary health centre and subcentre; (iii) identifying members from involved health disciplines who should be included in the upcoming outreach visits; (iv) optimizing the use of the institute’s resources; (v) reviewing the patient referral protocol; and (vi) brainstorming on feasible preventive care measures. 

The multidisciplinary rehabilitation team from the institute included two community medicine doctors, two gynaecologists, two paediatricians, one general physician, one orthopaedic surgeon, and one resident each from otorhinolaryngology, ophthalmology and dermatology departments. The multidisciplinary team also included a physiotherapy team comprised of one faculty member, one master’s student, one PhD student and five bachelor’s students from Mahatma Gandhi Mission School of Physiotherapy. The team’s task was to visit the primary health centre, subcentre and villages twice per week to address the health and rehabilitation needs of the population. 

The institute also organized a multidisciplinary programme to address the identified health-pertinent issues during the rural appraisal. The programme consisted of: (i) health camps delivered by the multidisciplinary team including the physiotherapy team, who clinically evaluated pregnant women and managed identified health concerns; and (ii) community health education programmes. 

**Fig. 1:**
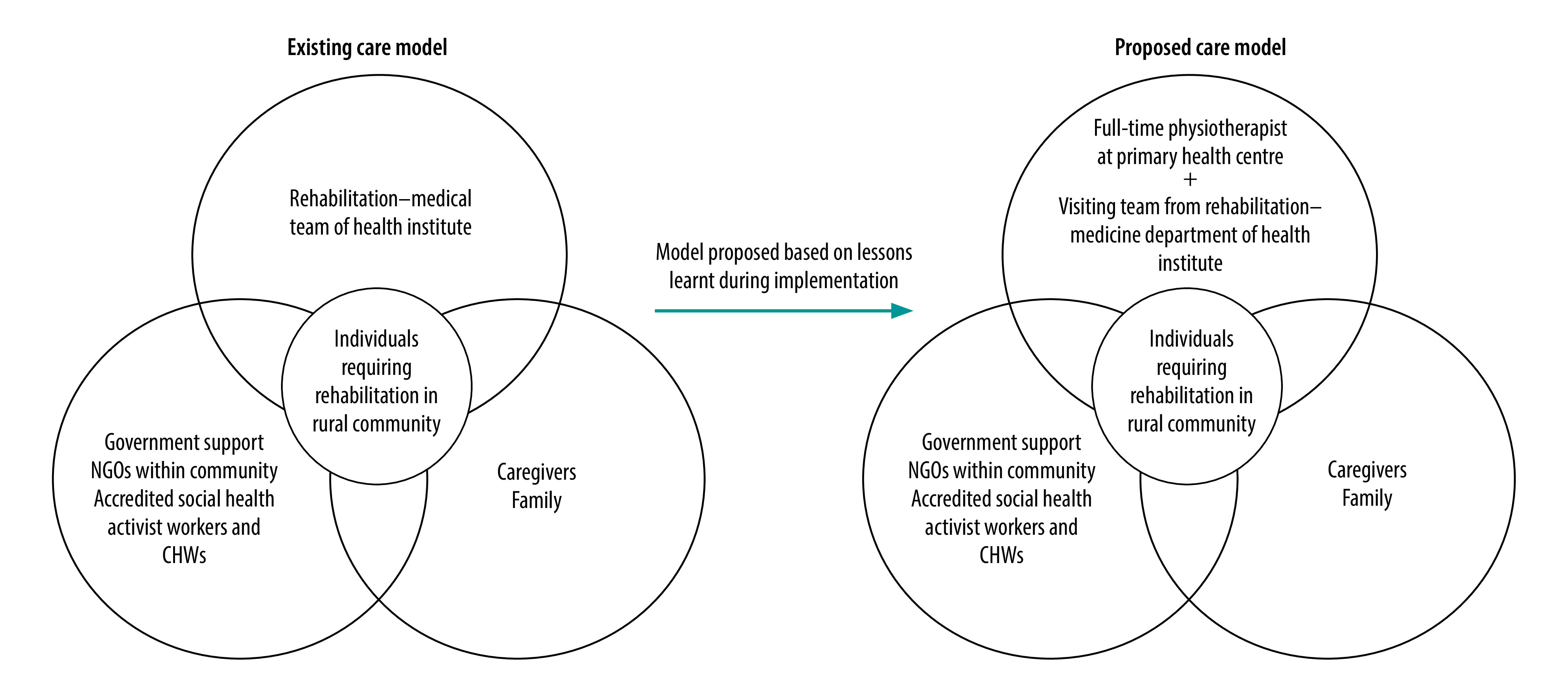
Existing and proposed model of care adopted at Mahatma Gandhi Mission Institute of Health Sciences to integrate rehabilitation into primary care, India, 2018–2021

CHW: community health worker; NGO: nongovernmental organization.

This service delivery model used qualified workforce and resources available at Mahatma Gandhi Mission Institute of Health Sciences to strengthen existing government efforts (Fig. 1). The institute developed this model in alignment with the recent updates in national health-care policy,^4^ which attempts to address the growing burden of noncommunicable diseases; reduce unsustainable health expenditure; fill gaps in infrastructure in health-care facilities; and address scarcity of CHWs and community rehabilitation personnel, especially in rural areas.^11^ Gaps in implementation of existent policies, especially in the area of health promotion, prevention and management of commonly reported health conditions, led to the development of the service delivery model. 

### Implementation

In 2018, the physiotherapy team conducted door-to-door surveys in households and screened all the present villagers for major health conditions, such as musculoskeletal pain, neurological conditions, cardiorespiratory conditions, and women’s health issues such as stress urinary incontinence and urogenital prolapse. Following the survey, patients requiring rehabilitation were referred to the primary health centre for physiotherapy. The referral protocol instructed the physiotherapy team to refer patients with a medical history indicating a risk of having a serious disorder, such as infection, cancer or fracture,^12^ to Mahatma Gandhi Mission University Teaching Hospital, Kamothe for tertiary-level multidisciplinary care.

Household members identified with rehabilitation needs received information about the outreach visits. CHWs reminded the patients about these visits a day before the planned visits through public announcements on a loudspeaker. 

The multidisciplinary team made day-long (8 hours) outreach visits to the primary health centre and Dhamani subcentre twice a week for the delivery of rehabilitation services for patients identified during the survey. On the first visit to the primary health centre, patients were attended to on a first-come first-served basis. The services were free of charge to all patients. Patients with identified health conditions were given a follow-up appointment at the next outreach visit. CHWs reminded patients about follow-up visits through public announcements on a loudspeaker. We used existing resources of the institute to deliver rehabilitation services, and the institute supported the costs for the travel of the team and for portable equipment.

For the health camps, the multidisciplinary team used the *Surakshit Matritva Din* platform offered by *Pradhan Mantri Surakshit Matritva Abhiyan* to deliver antenatal care to pregnant women at the primary health centre once a month. This scheme is funded by the government to ensure comprehensive and quality antenatal care, free of cost across the nation on the ninth day of every month.^13^ The team prescribed medicines and nutritional supplements and provided physiotherapy. CHWs helped with dissemination of information about the camp and assisted with the registration and crowd management. Before the camps, the team obtained medicine and consumable supplies from the university teaching hospital. The institute supported the transportation of supplies. 

To discuss the logistics of creating awareness, review the activities conducted and strategies to garner community support, regular meetings were held every 6 months. These meetings included members of the *gram panchayat*, a public health nurse and a medical social worker of the community medicine team, and university teaching hospital administrators, and took place at the Maldung *gram panchayat* office or the university teaching hospital.

To build awareness regarding the role of physiotherapy in women’s health, we adopted strategies to reach women and children in need of rehabilitation by approaching them during antenatal health check-up and immunization campaigns at the primary health centre. Simultaneously, community health education programmes delivered by resident doctors and physiotherapy master’s students built awareness regarding the need for rehabilitation among villagers and CHWs. The team used various strategies such as health talks, awareness rallies, poster exhibitions and street plays, as well as the distribution of informative pictorial brochures, the display of educational charts, and the provision of exercises, yoga, ergonomic advice and home-based self-care strategies to empower patients through self-rehabilitation. 

## Results

Findings of the participatory rural appraisal and focus group discussions revealed lack of awareness, lack of health facilities at village level and in hamlets, lack of CHWs in one village, lack of medical doctors at the subcentre and lack of rehabilitation personnel in all five villages. The rural participatory appraisal also revealed health-pertinent issues such as infectious diseases, noncommunicable diseases and anaemia (Table 1). 

**Table 1:** Key problems identified and lesson learnt during integration of rehabilitation services into primary care in a rural setting, India, 2018–2021

Key problems identified^a^	Processes identified	Key findings	Lessons learnt
Lack of awareness regarding need for rehabilitation and available health-care solutions	• Villagers were introduced to the health-care team during survey, camps, health check-up, antenatal check-up and immunization drive conducted at primary health centre • Awareness talks by physiotherapists on specific health conditions • Distribution of informative pictorial brochures to patients and caregivers • Use of informative charts and street plays to highlight benefits offered by rehabilitation • Door-to-door screening for need assessment	• Increase in awareness regarding the need for rehabilitation in the management of various health conditions such as spine pain, knee pain, antenatal care, postnatal care, developmental disorders and stroke among people in the local setting • The screening survey identified the need for rehabilitation over the implementation period • 900 adults with musculoskeletal disorders • 180 infants, children and adults living with various neurological disorders • 1629 antenatal women with musculoskeletal pain and/or stress incontinence	• A specific in-depth need assessment for rehabilitation services is essential to plan required resources for uninterrupted care • This rehabilitation service delivery model can be used to initiate improvement in attitudes, correct beliefs about various noncommunicable diseases, maternal and child health, and developmental disorders, increase awareness regarding rehabilitation options and encourage people to seek care early on to minimize disability and optimize functioning
Inadequate qualified multidisciplinary rehabilitation workforce and lack of access to care	• Strengthened workforce in the community through using resources of the university teaching hospital, namely experts in community medicine, physiotherapy, orthopaedics, gynaecology and other disciplines, faculty members, postgraduate students, interns and residents • Use of university transport to reach distant locations of rural villages to offer rehabilitation services	• The model is sustainable as the workforce resources of the health institute are available consistently • All specialty services provided by the multidisciplinary rehabilitation team exist at the health institute • Faculty members are experienced in evidenced-informed care • A large taskforce of postgraduate students and interns are involved in delivery of care enabling a greater reach-out • Students can connect with local problems faced by the community and are exposed to real-life situations which instil critical thinking for problem solving • Students feel a sense of societal contribution • Patients do not need to travel long distances to access health care, therefore the model is economically viable for patients	• Workforce reconfiguration with inclusion of dedicated and qualified multidisciplinary rehabilitation team members and offering services within the community to increase the use of rehabilitation services
Lack of infrastructure and resources at primary health centre and subcentre	• We encouraged use of tools available in local setting, e.g. use of mud packs for local heat application in patients with knee pain	• Use of portable resources of university teaching hospital to offer rehabilitation services in primary care	• Resources of the university teaching hospital can be used to initiate care at primary level. • Infrastructure needs can be met by engaging in research-driven activities, engaging the local governments and developing industry partnership

^a^ We identified the problems during a participatory rural appraisal and a door-to-door survey of villagers.

During the door-to-door survey, we questioned 2073 people about spine care. The point prevalence of low back pain was 4.9% (101 respondents) and neck pain was 2.9% (61 respondents). Among the 250 tribal people surveyed, 10% (25 respondents) had low back pain and 3.6% (9 respondents) had neck pain.^14^ Additionally, screening for developmental disorders revealed lack of knowledge among 500 new mothers about age-appropriate, neurodevelopmental milestones and need for postnatal exercises for new mothers.

To address the rehabilitation needs identified through the screening, the physiotherapy team offered physiotherapy care through the outreach visits. During the implementation, an average of 50 patients (range: 35–55) attended the outreach visits per month. In total, the physiotherapy team provided care for 1800 patients, of which 50% (900) of patients, including elderly people, presented with musculoskeletal disorders, such as knee pain and spine pain; 10% (180) with neurological disorders; and 40% (720) were women who received postnatal care during immunization campaigns. 

Between 2018 and 2021, 40 health camps were conducted, and the physiotherapy team delivered antenatal care to 1629 pregnant women with musculoskeletal pain or stress urinary incontinence. On the *Surakshit Matritva Din *platform, the team educated pregnant women about antenatal physiotherapy care, offered antenatal physiotherapy care, and then followed them up during consecutive outreach visits.

The multidisciplinary team offered rehabilitation services to all patients who reported disability due to musculoskeletal, neurological, cardiorespiratory or geriatric health problems or women requiring antenatal or postnatal care at primary care level. None of the patients required referral for tertiary care at the university teaching hospital. Of the 900 patients with musculoskeletal disorders who underwent rehabilitation, 360 (40%) performed daily-living activities and continued to work with reduced pain within 2–3 days after rehabilitation. 

In parallel, the pilot implementation of the service delivery model helped to sensitize and train 945 bachelor’s students and 67 master’s students of physiotherapy in service delivery at primary care level through a real-life problem-solving learning approach. 

The estimated cost for delivery of rehabilitation services per year was computed by accounting for the cost of human resources, capital resources including rental of the primary health centre building, medicine, transport, equipment, furniture, stationery and printing. Total in-house cost of delivery of rehabilitation care was 876 277 Indian rupees (about 11 033 United States dollars) per year. However, projected cost for rehabilitation service delivery in primary care by an external dedicated rehabilitation service agency is likely to be higher, as the current model was embedded within the resources of a multidisciplinary health institute.

## Discussion

Our experience of providing rehabilitation services in a rural part of India taught us several lessons (Box 1). The model of service delivery resulted in changes for various stakeholders, including patients and caregivers, the health institute, community volunteers and policy-makers. This approach helped to build knowledge and awareness about rehabilitation needs, leading to increased demand for physiotherapy services. The approach also helped to improve attitudes and beliefs of patients and caregivers, through first-hand experience of substantial improvement in functional status and well-being.

Box 1 **Summary of main lessons learnt**

- Rehabilitation service delivery offered by health institutes needs to be complemented by the rehabilitation workforce including full-time physiotherapist and visiting speech therapist, occupational therapist, prosthetist and orthotist at primary care facilities.
- The multidisciplinary rehabilitation workforce needs to be strengthened by partnerships with industry and nongovernmental organizations.
- To improve detection, rehabilitation and monitoring, new technology needs to be integrated into the service delivery model.

Raising awareness among policy-makers – such as the *gram panchayat*, government officers and local leaders – through focused group discussions and meetings, facilitated delivery of rehabilitation services. The experience gained during the implementation of the service delivery model helped to organize basic infrastructure for a full-time physiotherapist at the primary health centre using human resources and portable equipment from the institute. 

The service delivery model informed the institute’s strategic research planning framework. This framework guides the institute to design and conduct implementation research that identifies evidence-informed, culturally acceptable models of rehabilitation service delivery. Linking the model to student training at the institute made the model sustainable, both economically and workforce wise. The model was also used for engaging students in the communities and in social responsibility, by conducting community-based participatory research. Training students in real-life problem-solving learning approaches is pivotal in creating a future trained and sensitized rehabilitation workforce.

The service delivery model faced various challenges at the beginning of implementation such as community acceptability due to lack of knowledge about rehabilitation, the team’s access to remote areas and transport for the workforce. We gained community acceptability by adopting a participatory approach, wherein CHWs were engaged along with community leaders in sensitization of people (Table 1).

We anticipate that the magnitude of rehabilitation need in the district is higher because we were unable to survey the entire population of the five villages, as a few houses were closed at the time of the survey, some people were at work or migrated to other areas.^14^ Therefore, further need assessment is essential for precise planning of necessary resources for rehabilitation services. Alongside this, the service delivery model created a need for development of a central database spanning across district, state and national levels to monitor rehabilitation outcomes.

The service delivery implementation model faced disruptions caused by emergencies such as the coronavirus disease 2019 pandemic and heavy monsoon. The climatic conditions made access to the primary health centre and villages difficult. Other challenges causing disruptions were limited transport facility and time constraints caused by academic engagements of faculty members and students.

Therefore, we recommend making provision for a full-time dedicated physiotherapist at primary health centres supported by visiting rehabilitation personnel (Fig. 1). Furthermore, strengthening the model through partnerships with industry and nongovernmental organizations (NGOs), sharing similar ideologies, is essential to augment government resources. We are currently attempting to partner with NGOs working in this field and are seeking funding from the industry through corporate social responsibility initiatives.

In addition to the need for training caregivers and CHWs, formal engagement of people with disabilities into the planning and implementation of the rehabilitation service delivery model is imperative for the outcomes. 

To reach more people, integrating technology into service delivery is warranted. We are in the process of developing mobile-based applications to strengthen existing capacities for detection, rehabilitation and monitoring.

By involving the large network of government and private health institutes in India, the service delivery model presented here can be scaled up, leading to more people with rehabilitation needs improving their functional health. Subsequently, this improvement will contribute to the overall sustainable development of the rural health sector.

## References

[1] Magnussen L, Ehir i J, Jolly P. Comprehensive versus selective primary health care: lessons for global health policy. Health Aff (Millwood). 2004 May-Jun;23(3):167–76. 10.1377/hlthaff.23.3.167 15160814

[2] Kumar SG, Roy G, Kar SS. Disability and rehabilitation services in India: issues and challenges. J Family Med Prim Care. 2012 Jan;1(1):69–73. 10.4103/2249-4863.94458 24479007 PMC3893941

[3] Kamenov K, Mills JA, Chatterji S, Cieza A. Needs and unmet needs for rehabilitation services: a scoping review. Disabil Rehabil. 2019 May;41(10):1227–37. 10.1080/09638288.2017.1422036 29303004

[4] National Health Policy-2017. New Delhi: Ministry of Health & Family Welfare, Government of India; 2017: p. 3. Available from: https://www.nhp.gov.in/nhpfiles/national_health_policy_2017.pdf [cited 2022 Feb 26].

[5] Sanjeev S, Waingankar P, Anjenaya S, Lohare BS. A study of reasons for nonimmunization among children attending the services of a rural hospital in Raigad district, Maharashtra. MGM J Med Sci. 2016;3(2):57–61. 10.5005/jp-journals-10036-1090

[6] Kakumani KV, Waingankar P. Assessment of compliance to treatment of diabetes and hypertension amongst previously diagnosed patients from rural community of Raigad district of Maharashtra. J Assoc Physicians India. 2016 Dec;64(12):36–40. 28405986

[7] Bhakare SS, Mohanty N, Gopalkrishnan S. Effect of planned teaching on practices of skilled birth attendants on ‘facility based newborn care’ at health care facilities in Raigad district, Maharashtra. Int J Health Sci Res. 2017;7(5):232–42. Available from: https://www.ijhsr.org/IJHSR_Vol.7_Issue.5_May2017/37.pdf [cited 2022 Sep 15].

[8] Amonkar P, Mankar MJ, Thatkar P, Sawardekar P, Goel R, Anjenaya S. A comparative study of health status and quality of life of elderly people living in old age homes and within family setup in Raigad District, Maharashtra. Indian J Community Med. 2018 Jan-Mar;43(1):10–3. 10.4103/ijcm.IJCM_301_16 29531431 PMC5842466

[9] District census handbook. Raigad: Directorate of Census Operations Maharashtra; 2011.

[10] Abhijit B. Health and nutritional status of tribal children in Raigad district of Maharashtra. IJDTSW. 2015 Jun;3(1):31–65. Available from: http://www.ticijournals.org/health-and-nutritional-status-of-tribal-children-in-raigad-district-of-maharashtra/ [cited 2022 Feb 27].

[11] Pati MK, Swaroop N, Kar A, Aggarwal P, Jayanna K, Van Damme W. A narrative review of gaps in the provision of integrated care for noncommunicable diseases in India. Public Health Rev. 2020 May 13;41(1):8. 10.1186/s40985-020-00128-3 32435518 PMC7222468

[12] Finucane LM, Downie A, Mercer C, Greenhalgh SM, Boissonnault WG, Pool-Goudzwaard AL, et al. International framework for red flags for potential serious spinal pathologies. J Orthop Sports Phys Ther. 2020 Jul;50(7):350–72. 10.2519/jospt.2020.9971 32438853

[13] Abhiyan PMSM. New Delhi: Government of India, Ministry of Health & Family Welfare; 2016. http://www.nrhmhp.gov.in/sites/default/files/files/PMSMA-Guidelines.pdf [cited 2022 Sep 15].

[14] Mullerpatan R, Nahar S, Singh Y, Cote P, Nordin M. Burden of spine pain among rural and tribal populations in Raigad District of Maharashtra State of India. Eur Spine J. 2021 Apr;30(4):1004–10. 10.1007/s00586-020-06585-3 32914232

